# Implementing Monocular Visual-Tactile Sensors for Robust Manipulation

**DOI:** 10.34133/2022/9797562

**Published:** 2022-09-05

**Authors:** Rui Li, Bohao Peng

**Affiliations:** School of Automation, Chongqing University, Chongqing, China

## Abstract

Tactile sensing is an essential capability for robots performing manipulation tasks. In this paper, we introduce a framework to build a monocular visual-tactile sensor for robotic manipulation tasks. Such a sensor is easy to manufacture with affordable ingredients and materials. Based on a marker-based detection method, the sensor can detect the contact positions on a flat or curved surface. In the case study, we have implemented a visual-tactile sensor design specifically through the framework proposed in this paper. The design is low cost and can be processed in a very short time, making it suitable for use as an exploratory study in the laboratory.

## 1. Introduction

Tactile is an essential information source to improve the performance of planning [1] and control [2] for a robotic manipulator, so as to achieve complex robotic manipulations [3]. It characterizes the local contact and force relationship between the object and the hand. In human hands, there are numerous mechanoreceptive units (mechanoreceptors), making the human hands very sensitive to a variety of contact information [4, 5], which is the basis for dexterous manipulation, such as shape estimation [6] and origami folding [7], and would possibly be applied to applications such as home service and surgery [8, 9].

Although there have been various principles to achieve a tactile sensor [10], the visual-tactile sensor is becoming a practical means of implementing tactile sensors, since vision is no doubt a low-cost and effective source to provide abundant information. In recent years, owing to the significant advances in visual information processing, the use of vision to build tactile sensors has become one of the concerning areas of the research community.

The visual-tactile sensor (VTS) can be traced back to 2004, when Kamiyama et. al [11] proposed visual marker detection based tactile sensor design, which characterizes the deformation of the elastic layer of the tactile sensor by the displacement of the markers. Subsequently, a series of studies and extensions of marker-based visual-tactile sensors (MVTSs) were conducted. Later, the retrographic sensing technique was proposed [12], and the high-resolution deformation of the elastic layer can be retrieved using oblique illumination.

**Figure 1:**
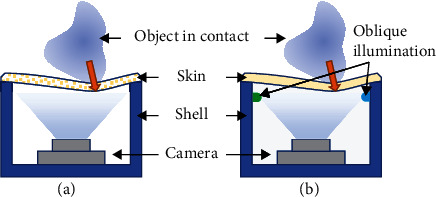
The general structures of visual-tactile sensors. (a) The marker-based visual-tactile sensor (MVTS). (b) The retrographic sensing-based visual-tactile sensor (RSVTS).

Presently, the main visual-tactile sensor principles can be divided into marker-based and retrographic sensing-based visual-tactile sensors (RSVTSs), as shown in Figure 1. The MVTS is very easy to build due to its simple structure and lack of strict light source requirements. Representative works include GelForce [11, 13], FingerVision [14], TacTip [15], and GelStereo [6, 16]. Its accuracy depends mainly on the density of the markers and the minimum resolution at which the camera can recognize the markers. Spherical markers are usually adopted for easy detection. The key point is the design of the markers and the acquisition of the spatial location of each marker. For instance, GelForce constructs markers in two different layers and observes their relative displacement under deformation to obtain contact and force information. TacTip utilizes the built-in spatial structure of the markers to obtain surface deformation. The GelStereo obtains the spatial position of each marker point based on the binocular pair polar geometry. Since the marker features are easy to detect in most cases, the elastic layer can be made transparent so that the camera can see through it while maintaining the detection accuracy, thus extending the usability of such sensors (e.g. object detection and proximity detection).

The RSVTS, due to retrographic sensing techniques, can make full use of the full camera pixels to obtain high-resolution deformation at the elastic layer. Representative works include GelSight [17, 18], GelSlim [19, 20], GelTip [21, 22], and DelTact [23]. The key point is the acquisition of photometric stereo. For example, GelSight/GelSlim/GelTip acquire tactile membranes through light sources installed at different locations. Dong et al. [18] optimized the illumination system of the original GelSight [17] so as to improve its accuracy and resolution. GelTip extends the surface of the elastic layer to domed shape. DelTact converts the setup of a lighting system to a dense color pattern elastic layer, whose pattern can be tracked by the optical flow algorithm with high resolution without interpolation. Since such devices require an undisturbed lighting system, the camera and the light in such devices are usually enclosed by a chamber environment.

Current work has yielded many encouraging results, but there is still room for improvement of visual-tactile sensors in the following aspects: (1) VTSs are generally easy to fabricate compared to non-VTSs. Many offer open-source designs, such as TacTip [15], GelSlim 3.0 [20], and DIGIT [24], but the lighting system, the elastic layer, and the customized sensing component still make replication of these designs costly. (2) Most of the VTSs adopt a flat contact surface, which makes them less competitive than their non-VTS counterparts. Supporting curved contact surfaces would be one of the major advantages of VTSs. (3) To take full advantage of the sensing capability of the visual sensors, the contact surface should preferably be of a transparent design to provide a basis for obtaining information not only on the elastic layer but also beyond the layer. Therefore, the motivation of this paper is to provide a framework for designing low-cost, easy-to-build visual-tactile sensors and to obtain general ideas for the design of such sensors.

The main contributions of this paper are as follows:

1. We propose a pipeline to prepare the elastic layer for the contact surface of the visual-tactile sensor. Through this pipeline, a curved, multilayered elastic surface can be personalized
2. We propose a marker-based contact position estimation method that can detect multiple contact regions simultaneously

The remaining sections of this paper are organized as follows: Section 2 introduces the framework scheme for the design of the visual-tactile sensor.  Section 3 describes the preparation pipeline of the elastic layer.  Section 4 illustrates the general use of this framework through a case study.  Section 5 concludes and discusses future work.

## 2. Framework Scheme

### 2.1. Overview

**Figure 2:**
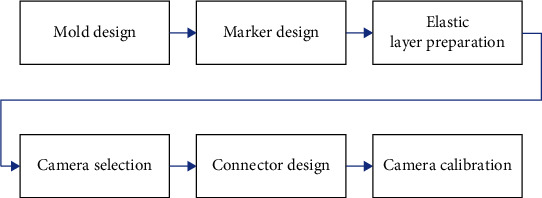
The overall framework to build a visual-tactile sensor.

To ensure that the designed visual-tactile sensor is low cost and easy-to-build, it is necessary to keep the number of components of the sensor as small as possible and to keep the difficulty of preparing each component as little as possible (Figure 2). To meet this requirement, the proposed sensor consists of only three main components: the elastic layer, the camera, and the connectors.

The elastic layer is what is referred to as “skin” and where the contact between the object and the sensor occurs. Once the object is pressed against the elastic layer, geometric deformation will take place. The camera is the core component used to sense the deformation of the elastic layer via the changes in the captured images. The connectors are used to combine the elastic layer with the camera and then fix them to the manipulator.

### 2.2. Elastic Layer Design

The elastic layer is where contact occurs. With long-term use, this component will inevitably wear and age. Therefore, materials with characteristics such as wear resistance, ease of preparation, ease of replacement, and low cost should be considered. In a simple laboratory environment, materials that can be processed at ambient or easily achievable temperatures should be selected so that the elastic layer can be replaced easily and quickly when needed.

We selected two common and easily accessible materials: silicone and epoxy resins, as illustrated in Table 1. These two materials are versatile and are commonly used to process a variety of flexible parts.

In this work, we prefer to use silicone resin as the elastic layer material. Although the above two resins share a similar appearance when prepared, epoxy resin will turn yellow in the UV environment and will gradually harden with time, while silicone resin will not suffer from these disadvantages.

### 2.3. Camera Selection

It requires that we choose the camera module with the smallest possible size, wide viewing angle, and short focal length, due to the need to miniaturize the entire visual-tactile sensor.

The images captured by monocular cameras lose scale information, i.e., the distance of each pixel point to the origin of the camera coordinate system cannot be obtained. Although the problem of depth estimation can be solved by introducing binoculars, such modules will increase the size of the vision sensing part, and the binocular systems usually need a very consistent sensor and lens, thus requiring careful calibration, which will undoubtedly increase the cost and production difficulty of the system. In this sense, the introduction of a binocular system is contrary to the motivation of this work. Therefore, a monocular camera will be used in this framework. Scale information can be obtained by detecting markers with known dimensions.

### 2.4. Connector Design

The connector is used to integrate the elastic layer and the camera. This component should be kept as small as possible to reduce the overall size of the visual-tactile sensor. 3D printing would be the best choice to create the connector.

## 3. Pipeline of the Elastic Layer Preparation

To prepare the elastic layer, we propose the following pipeline: step 1: design the mold. The shape of the mold determines the shape of the elastic layer. Step 2: design the marker. The marker affects the performance of deformation detection. Step 3: manufacture the elastic layer. A series of processes, such as proportioning, mixing, removing air bubbles, and curing, will be taken to obtain the desired elastic layer.

### 3.1. Mold Design

**Figure 3:**
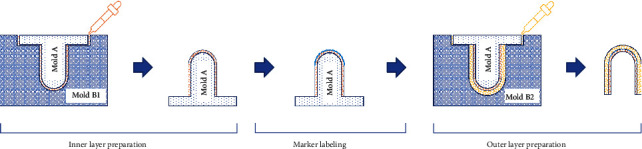
The overall framework to build a visual-tactile sensor.

We use an injection molding process to prepare the elastic layer, which is very easy to implement in a laboratory environment. We simply need to create mold *A* and mold *B* as shown in Figure 3 to form the elastic layer in the desired shape.

Depending on the elastic material used, a suitable mold material needs to be selected to prevent the elastic layer from not being released after curing. The elastic layer should be composed of at least two sublayers, the inner layer and the outer layer, the middle of which is used to label markers. To achieve that, we can design several mold *B*s (*B*_1_, *B*_2_, ⋯) and prepare the sublayers one by one, from the inner sublayer to the outer sublayer.

It is worth noting that it is necessary to create necessary holes to let the liquid flow out of the mold, when the volume of the liquid changes during the curing process, thus maintaining the expected shape of the elastic layer.

### 3.2. Marker Design

**Figure 4:**
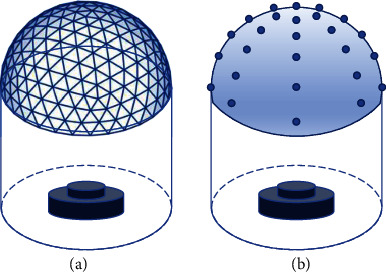
The layout of the markers when the elastic layer is in a domed shape. (a) The markers will be filled within every triangle of the *N* frequency geodesic dome. (b) The markers are plotted with equal distances in spherical coordinates.

The shape of the markers can be either primitive or fiducial markers. To obtain the deformation of the elastic layer, the depth of the marker position should be estimated either by knowing the dimensions of the marker or by constructing multiple layers of markers and estimating them by their relative offsets. As shown in Figure 4, when the elastic layer is dome-shaped, the markers can be arranged either (a) by generating an *N* frequency geodesic dome with the markers arranged in the center of each triangle or (b) by uniformly separating the markers in spherical coordinates or (c) by any customized layouts.

### 3.3. Elastic Layer Manufacturing

The manufacturing process of the elastic layer is shown in Figure 3. First, we mix the base and the curing agent of the liquid material used in the desired ratio and inject the mixture into mold *B*_1_ through the dropper. Then, we cover mold *A* to mold *B*_1_ and wait for the mixture to cure. After curing, we remove the inner layer from mold *B*_1_ and label the markers on the outer surface of the inner layer. Finally, we prepare the mixture again and inject it into mold *B*_2_, cover mold *A* with the inner layer onto mold *B*_2_, and wait the mixture to cure. After curing, we separate mold *A* and mold *B*_2_ from the elastic layer.

## 4. Case Study

In this section, we follow the framework proposed in Section 2 to build the sensor. The elastic layer is designed as finger-sized and finger-shaped, so that it can be equipped with robotic fingers and the whole fingertip can perceive tactile information.

### 4.1. Elastic Layer Preparation

Our goal was to create a tactile sensor that is similar in length and size to the distal plus middle phalanges of the human finger. According to [25, 26], the general length range of the human distal plus middle phalanges is between 18.9 and 40.3. Therefore, in this paper, an elastic layer of the visual-tactile sensor with length 25.0 is designed.

To keep the shape of the elastic layer as simple as possible, the elastic layer consists of two parts, the dome and the cylinder. For the preparation of the elastic layer, a mold was made from aluminum. The thickness of the elastic layer is determined by the inner and outer diameters of the mold, in this case, mold *A* with a diameter of 8.0 and mold *B* with diameters of 10.0 and 12.0, respectively.

**Figure 5:**
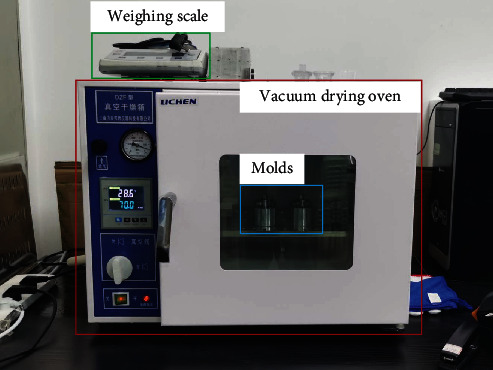
The vacuum drying oven, the weighing scale, and the molds.

**Figure 6:**
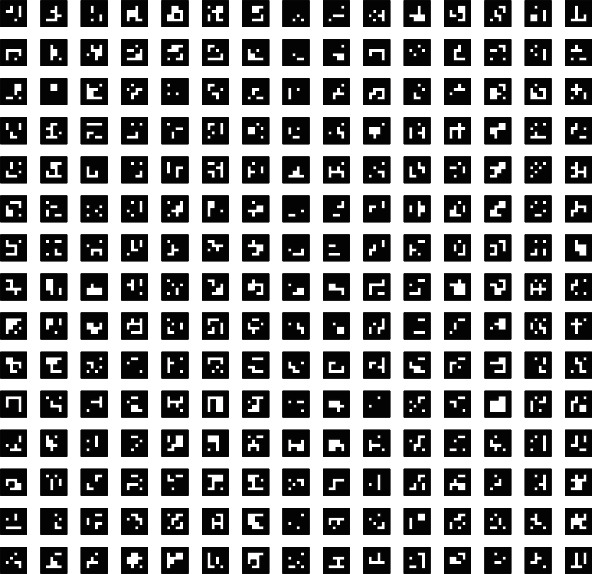
The ArUco markers used in this case study.

In this case study, we used silicone resin as the material to make the elastic layer. Specifically, we used SYLGARD 184 in our experiment. We use a vacuum drying oven and a weighing scale (as shown in Figure 5) to make the preparation process accurate and repeatable. The specific preparation process was as follows:

1. The base is mixed with the curing agent in proportion. The ratio of base to curing agent is shown in Table 2. As the material itself is viscous, the mixed liquid needs to be put into a vacuum drying oven, pumped to a value of -0.1, and then left to stand for 40 to expel any air bubbles in the liquid, to obtain a clear mixture
2. Mold *B*_1_ is covered onto mold *A* and placed in the vacuum drying oven, heated to 70, and vacuumed for curing. The curing process takes approximately 40, after which the molds can be removed once the vacuum drying oven has cooled down and the air pressure has been restored. After unscrewing, the inner elastic layer is carefully removed from the mold *B*_1_
3. The marker is then added to the outer surface of the inner elastic layer. In the case study, we print the markers on polyethylene terephthalate (PET) transparent label stickers and place them onto the surface with a customized layout as illustrated in Figure 6
4. Mold *B*_2_ is covered onto mold *A* and placed in the vacuum drying oven, heated to 70, and vacuumed for curing. The curing process takes approximately 40, after which the molds can be removed once the vacuum drying oven has cooled down and the air pressure has been restored. After unscrewing, the whole elastic layer can be carefully removed from mold *B*_2_ to mold *A*

### 4.2. Camera Selection and Marker Design

To obtain a clear, complete view of all the markers inside the dome, we chose a compact, short focal length, wide-angle monocular camera. The camera costs approximately 23. We used a checkerboard grid method to calibrate the camera and correct the distortions.

In the case study, we use ArUco [27, 28], a binary square fiducial marker, to illustrate the idea of marker detection and deformation estimation. The main advantage of fiducial markers is that they are simply flat patterns but convey three-dimensional information.

To use the ArUco markers, the following steps are adopted:

1. *Marker creation*: a predefined ArUco marker dictionary DICT_4X4_250 is chosen since it can provide 250 distinct markers, which makes it easy to identify each marker with a unique ID. Each of the markers takes at least 6 × 6 = 36 pixels only so that they can be printed small but can still be detected with ease
2. *Layout of the markers*: in this case study, we use a customized layout to arrange the markers. Compared to the spherical coordinate layout, the markers are geometrically equidistant and better reflect the deformation of the elastic layer equally. The markers are printed with a size of 1.5 × 1.5
3. *Marker detection*: images with markers are captured by a monocular camera. To robustly and stably detect ArUco markers, we preprocess the images captured by the monocular camera with image enhancement and binarization methods. An additional filter is then applied to reduce light interference and noise, improving the detection efficiency. Afterwards, we draw a frame along the edge of each marker and calculate the center coordinate. Subsequently, a PNP (perspective *n*-points) mapping equation is constructed based on the intrinsic matrix and distortion parameters of the camera, as well as the physical width of the marker

**Figure 7:**
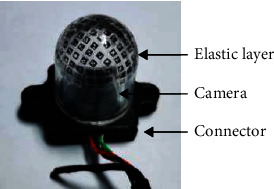
The implemented visual-tactile sensor in this case study.

Finally, we 3D print a connector to integrate the elastic layer with the camera. The implementation is shown in Figure 7.

### 4.3. Contact Position Estimation

In this case study, the position of the contact point can be estimated as follows: First, an image of the elastic layer at rest is obtained, and the positions *p*_*i*_ of the marker point on the image are detected; then, after the elastic layer has deformed, the positions ${\hat{p}}_{i}$ of the markers on the image are detected again; for each marker, we can obtain the magnitude of displacement $\Delta p_{i}=\left|p_{i}-{\hat{p}}_{i}\right|$ of that marker before and after the deformation. An image with the same resolution of the original image is redrawn. The intensity of each pixel ranges from 0 to 1. A series of gradient filled circles is drawn in this image. The center of each circle is *p*_*i*_, and the pixels at a distance *l* from the center of the circle have an intensity value *I*(*l*) = *l*/Δ*p*. If a pixels falls in several circles, the gray values are superimposed (and the maximum value does not exceed 1). The watershed algorithm is then used to obtain local extreme value regions, the centers of which are the contact points.

## 5. Conclusions

In this paper, a framework to build a low-cost, monocular visual-tactile sensor is proposed. It can detect the contact positions on a flat or curved surface, providing a comprehensive perception area. We also introduced a method to estimate the contact positions. The design is low cost and can be processed in a very short time, making it suitable for use as an exploratory study in the laboratory.

In the future study, we will focus on the improvement of resolution by designing novel marker patterns. And we will explore the use of this sensor in applications such as home services.

## Figures and Tables

**Table 1:** Specifications of silicone resin and epoxy resin.

Material name	Polydimethylsiloxane (silicone resin)	Polyepoxides (epoxy resin)
Typical product	Dow SYLGARD 184	Hexion Epon 828
Price	$61.22/kg	$49.82/kg
Transparent	Yes	Yes
Curing time	~35 min at 100°C	~2 h at 120~150°C
Disadvantages	Very viscous. The ratio of the base to the curing agent needs to be controlled very precisely.	Becomes discolored and hard over time

**Table 2:** Weight and ratio of the gradient in the case study.

Gradient	Base	Curing agent
Weight (g)	3.61	0.4525
Ratio	Approximately 7.98 : 1	

## Data Availability

The data used to support the findings of this study are available from the corresponding author upon request.
